# The Neural Basis of a Cognitive Function That Suppresses the Generation of Mental Imagery: Evidence from a Functional Magnetic Resonance Imaging Study

**DOI:** 10.3390/vision8020018

**Published:** 2024-04-10

**Authors:** Hiroki Motoyama, Shinsuke Hishitani

**Affiliations:** 1 College of Humanities and Social Sciences, Ibaraki University, Mito 3108512, Japan; 2 Emeritus Professor of Psychology, Hokkaido University, Sapporo 0600810, Japan; hishitani@gol.com

**Keywords:** mental imagery, visual perception, vividness, richness, suppression, posterior cingulate gyrus

## Abstract

This study elucidated the brain regions associated with the perception-driven suppression of mental imagery generation by comparing brain activation in a picture observation condition with that in a positive imagery generation condition. The assumption was that mental imagery generation would be suppressed in the former condition but not in the latter. The results show significant activation of the left posterior cingulate gyrus (PCgG) in the former condition compared to in the latter condition. This finding is generally consistent with a previous study showing that the left PCgG suppresses mental imagery generation. Furthermore, correlational analyses showed a significant correlation between the activation of the left PCgG and participants’ subjective richness ratings, which are a measure of the clarity of a presented picture. Increased activity in the PCgG makes it more difficult to generate mental imagery. As visual perceptual processing and visual imagery generation are in competition, the suppression of mental imagery generation leads to enhanced visual perceptual processing. In other words, the greater the suppression of mental imagery, the clearer the presented pictures are perceived. The significant correlation found is consistent with this idea. The current results and previous studies suggest that the left PCgG plays a role in suppressing the generation of mental imagery.

## 1. Introduction

What processes are involved in the generation of mental imagery? Most imagery researchers agree that conscious mental imagery is represented in the image construction stage through transformations applied to information retrieved from long-term memory (LTM) [1,2,3,4,5,6]. Kosslyn (1980; 1994) referred to the image construction stage as the visual buffer, stating that images formed in that buffer on the basis of information from LTM are mental images, whereas images formed in that buffer on the basis of information from the outside world are visual perceptual images [2,7]. This definition suggests that the image construction stage is a common process within mental imagery and visual perceptual imagery processing. This claim has been supported by numerous studies showing a competitive relationship between the visual perceptual process and the generation of visual imagery [8,9,10,11,12,13,14,15,16,17,18,19,20,21,22]. This is because these two processes share a common process, such that carrying out one process disrupts the other, resulting in competition. If this is the case, then for us to act adaptively, we would need a mental function that suppresses one process by not forming mental images while visual perceptual images are actively being formed, or vice versa, such as not forming visual perceptual images while mental images are being formed. Kosslyn (1987) suggested that visual hallucinations are produced when memory inputs from LTM to the visual buffer are strong enough to overwhelm perceptual inputs. If this is correct, then a function that suppresses imagery generation so as not to produce visual hallucinations may be active as we construct the visual world in everyday life [23]. The current study investigates the neural basis that directly supports the existence of this perception-driven imagery suppression mechanism.

Motoyama and Hishitani (2016) demonstrated the imagery suppression mechanism in a research context that was slightly different from that used in the present study [24]. They conducted an experiment to locate brain regions that are associated with the suppression mechanism for negative imagery generation. Previous studies have reported that the vividness of mental imagery varies according to the emotional valence of the imagined object, such that negative imagery is more vague than positive imagery [24,25,26]^1^. Participants in the above studies were simply asked to intentionally generate positive or negative imagery, but they were not instructed to intentionally control the vividness of the imagery, regardless of whether the imagery generated was positive or negative. Although the participants did not intend to generate vague negative imagery or vivid positive imagery, negative imagery was more vague than positive imagery. Hishitani (1995) and Hishitani et al. (2011) argued that, when the emotional valence of an object or an event is negative, imagery generation processing is suppressed because generating vivid negative imagery is stressful. As a result, the mechanism that suppresses negative imagery generation is activated during negative imagery generation, and negative imagery becomes more vague than positive imagery [1,27]^2^.

Motoyama and Hishitani (2016) attempted to identify the brain areas in which activation is significantly greater during negative imagery generation than during positive imagery generation, as they expected the suppression of mental imagery generation to be more active during negative imagery generation than during positive imagery generation [24]. They first showed that imagery generated in a positive condition was rated as more vivid than imagery generated in a negative condition. They then showed significantly increased activation in a part of the left posterior cingulate gyrus (PCgG) during the negative imagery generation condition compared to the positive imagery generation condition. In addition, the activity in this region was negatively correlated with the vividness of the negative imagery generated. That is, the greater the activity in this region, the less vivid the images in the negative imagery condition. If the left PCgG functions as a suppressor of the generation of mental imagery, especially negative imagery, then the results would be consistent with the following explanation: An increase in PCgG activity during negative imagery generation indicates a stronger suppressive function in the negative imagery condition. Therefore, the higher the activity of this brain area, the lower the vividness of negative imagery. These findings suggest that some areas of the left PCgG are involved in the emotion-driven suppression of imagery, which prevents the generation of imagery to avoid negative emotions. Furthermore, there was no correlation between PCgG activity and the vividness of positive imagery. This was interpreted as meaning that positive imagery was not necessary for emotional avoidance and that emotion-driven imagery suppression did not occur.

If the left PCgG is involved in imagery suppression in general, then it would be activated not only during the formation of negative imagery but also during other activities that require the suppression of the formation of mental imagery. That is, it is expected that at least some regions of the left PCgG would be activated while a person looks at visual perceptual images because of the competitive relationship between visual perceptual processing and visual imagery generation, as discussed above. Therefore, this study examines whether brain areas in the left PCgG are more activated in a visual object observation condition than in a positive imagery generation condition, in which mental imagery generation is considered not to cause, or mostly not to cause, the emotion-driven suppression of imagery. If there was a significantly activated area in the left PCgG, then it would provide evidence that this brain area is involved in the suppression of imagery generation during visual perceptual processing, i.e., the perception-driven suppression of imagery generation. It might be suggested that the resting period, during which there appears to be no mental activity, should be used as a control condition instead of the positive imagery generation condition. However, the rest period is not appropriate to be used as a control condition in this study. The PCgG is part of the default mode network that is activated at rest. At present, it is not possible to distinguish whether the activity observed in the left PCgG at rest reflects the automatic suppression of imagery generation or mental activity other than imagery suppression. The control condition in this study requires the following two conditions: (1) a picture is shown to provide the same perceptual information as in the picture observation condition but not to activate the perception-driven suppression of imagery, and (2) the emotion-driven suppression of imagery does not function. Therefore, it is considered an appropriate control condition to simply see a presented picture but not to perceptually process it and to generate positive imagery that is not the same as the presented picture.

In the picture observation condition in this study, a participant looks at a presented picture and evaluates its richness, i.e., the degree of clarity of the presented picture. Assuming that some brain areas in the left PCgG would be activated in the picture observation condition compared to in the control condition mentioned above, it could be speculated that the activated brain area plays a role in the perception-driven suppression of imagery. In this case, it is expected that there would be a correlation between the activity in the brain area and the participants’ richness ratings, which represent the subjective vividness of the presented picture in the picture observation condition. Several previous studies have shown that visual imagery acts as a disturbance in the detection and perception of presented visual stimuli [1,8,21,28]. Therefore, if the activated brain area in the left PCgG is active, then imagery generation processing would be suppressed, and visual perceptual processing, which competes with mental imagery processing, would be facilitated. In other words, if the left PCgG is more active, then the presented visual object would be perceived as being clearer because less mental imagery would be generated. Conversely, if the activity in the left PCgG region is weaker, then the presented visual object would be perceived as being more vague. If the above correlation between brain area activity and participants’ richness ratings is obtained, then it would be evidence that the activated brain area in the left PCgG plays a role in the perception-driven suppression of imagery generation.

Furthermore, if the activated brain area plays a role in imagery suppression, then some might think that there would be a negative correlation between the activation of the brain area and imagery vividness ratings. However, this is not the case in this experiment. This is because, firstly, the imagery generation condition in this study is set up as a condition in which the emotion-driven suppression caused by negative emotions is not, or almost not, produced. Secondly, because participants are not required to actively process the visual perceptual stimuli of the outside world, the perception-driven suppression of imagery will not occur during positive imagery generation. Therefore, there will be no correlation between the activation of the relevant brain area and the vividness of the imagery.

This study tested the following two hypotheses: (1) compared to the positive imagery condition, in which participants see a picture and generate imagery unrelated to it, stronger activity in the left PCgG is observed when participants observe visual perceptual information in the outside world without generating imagery (picture observation condition), and (2) there is a correlation between activity in the left PCgG and richness ratings. In addition, this study tested the hypothesis stating that there is no correlation between the vividness of positive imagery and left PCgG activity.

## 2. Materials and Methods

### 2.1. Participants

Healthy right-handed volunteers (*n* = 16, 5 females and 11 males with an average age of 26.8 years, SD = 5.7, ranging from 21 to 45 years) participated in this study, and each was paid 2000 JPY ($20) for their participation. The participants were undergraduate or graduate students at Hokkaido University. The Ethics Committee of Hokkaido University, Graduate School of Letters, approved the experimental protocol of this study. In addition, all participants gave written informed consent before participating. The handedness of the participants was assessed using the Japanese translation of the Edinburgh Handedness Survey [29], which indicated that all the participants were right-handed.

### 2.2. Materials

The words used to generate imagery in the positive imagery generation condition (Appendix A), which were not expected to activate the emotion-driven suppression of imagery function, were positive words that were used in [24]. The emotional valence of these words was based on the norms in [30], in which the emotional valence of a noun is rated on a 7-point Likert-type scale (where 1 indicates extremely negative emotions, and 7 indicates extremely positive emotions). The mean emotional valence of the words used in the positive imagery generation condition was 5.12 (ranging from 4.63 to 5.67), which was significantly positive compared to the neutral value, corresponding to a score of 4 (*t*(15) = 14.704, *p* < 0.00001). To prepare the pictures (Appendix B) presented in the positive imagery generation condition, nouns were selected from neutral words with an approximate neutral value of 4 (the mean emotional valence was 4.09, ranging from 3.92 to 4.24) in [30].

In the visual perceptual processing condition, a picture was shown on a screen that the participants were asked to look at (picture observation condition). The pictures presented corresponded to the words (Appendix A) used in the positive imagery generation condition. In the positive imagery generation condition, a noun appeared on the screen, and the participants were asked to form a mental image of the noun’s referent while seeing a picture. The pictorial stimuli (Appendix A and Appendix B) that were presented in both conditions were selected from ART EXPLOSION 750,000 Macintosh edition [31]. A Gaussian filter was applied to the prepared pictures at 4 levels (Gaussian effect 0 mm radius: no filtering, 4 mm, 8 mm, and 12 mm) by using Adobe Photoshop CS4, and the clarity of the presented pictures was varied. The higher the Gaussian effect, the greater the effect of the low-pass filter and the lower the clarity of the picture.

### 2.3. Apparatus

SuperLab Pro 4.4 (Cedrus Corporation, San Pedro, CA, USA) running on an Apple MacBook computer was used to present the stimuli and record the participants’ responses. An MRI-compatible response box [32] was used to record the participants’ subjective ratings of the vividness of the mental imagery and the richness of the pictures.

### 2.4. Procedure

**Figure 1:**
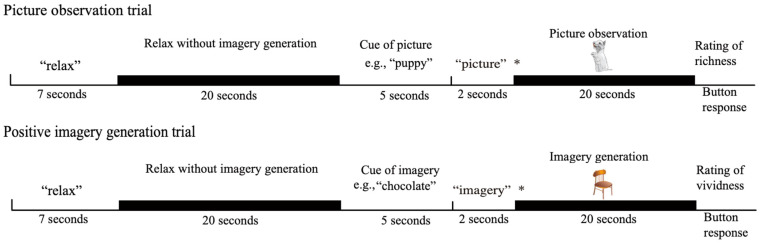
The experimental procedure: The asterisk (*) represents the cue for the participant’s task for the next 20 s. “Picture” indicates that their task was to observe the presented picture. “Imagery” indicates that their task was to generate imagery of the cue while seeing at the presented picture. The pictures presented in the picture observation and positive imagery generation conditions were, for example, a puppy and a chair.

The procedure used in this study included two activation conditions and a baseline condition (Figure 1). In the picture observation condition, the participants actively observed visual perceptual information in the external world without generating any imagery, whereas in the positive imagery generation condition, they performed a mental imagery generation task while seeing a picture. In this condition, the participants were asked to generate imagery while ignoring the presented picture. In the baseline condition, the participants were asked to relax without generating any imagery or looking at pictures.

If the participants’ task in the positive imagery generation condition had been to generate positive imagery without the input of any visual perceptual information from the outside world, then the brain activity during positive imagery generation would have been measured without the participants seeing a picture, whereas that during picture observation would have been measured with the participants looking at a picture. Therefore, not only would the presence or absence of the activity of the perception-driven imagery suppression function differ between the two conditions but the presence or absence of the input visual perceptual information would also differ. In this case, even if there was a difference in PCgG activity between the two conditions, it would not be clear whether it reflected the activity of the perception-driven suppression of imagery or the processing of visual perceptual information. Therefore, visual perceptual information was input in both the positive imagery generation and picture observation conditions, and the participants were asked to generate imagery without actively observing it in the former condition and to actively observe it in the latter condition.

The participants viewed the screen through a mirror system that was mounted onto a head coil, and their eyes were open while the experiment was conducted. In each picture observation trial, the word “relax” appeared on the screen for seven seconds, followed by a grey blank screen with no visual stimuli that was presented for twenty seconds. The participants were instructed to relax during the presentation of the blank screen (baseline condition). Then, a noun appeared on the screen for five seconds, followed by the word “picture”, which appeared on the screen for two seconds, and then a picture of the previously presented noun, which appeared for twenty seconds. The participants were asked to look at the picture when the word “picture” was presented after the noun. A message then appeared on the screen asking the participants to rate the richness of the picture. The participants rated the richness of the presented picture on a 4-point scale ranging from 1 (*very vivid*) to 4 (*very vague*)^3^. The responses were recorded on a computer using a response box placed next to the participants’ left hand. The next trial started when the participants pressed any button on the response box. The positive imagery generation trials were identical to the picture observation trials until the noun was presented after the baseline condition. In the positive imagery generation trials, the word “imagery” appeared on the screen for two seconds after the noun disappeared, and an image unrelated to the previously presented noun appeared for 20 s. The participants were asked to generate imagery of the referent of the noun. They were also asked to experience the positive emotion evoked by the generated imagery in order to reduce the emotion-driven suppression of imagery function. On this occasion, the participants were asked to generate imagery by superimposing imagery on the presented picture rather than by making associations with it. For example, if the participants generated an image of a kitten while seeing a picture of a bus, they were asked not to form an image of a kitten sitting on the roof of the bus or in the bus but rather to form an image in which the kitten was superimposed on the picture of the bus so that the picture of the bus formed the background of the image. In this condition, the participants were asked to ignore the incoming visual perceptual information while the picture was presented. However, it was possible for the participants to look elsewhere on the screen, where the picture was not presented, rather than at the presented picture, when they were asked to overtly ignore the presented picture. In this case, no visual information was provided to the participants. Thus, the participants were given the above instruction. This instruction was given to facilitate a common cognitive activity between the two conditions of picture observation and positive imagery generation. Then, a screen appeared asking about the vividness of the imagery, and the participants were asked to rate the vividness of their mental imagery on a 4-point scale ranging from 1 (*very vivid*) to 4 (*very vague*). A diagram of the present study is shown in Figure 1. Each session consisted of eight trials (four picture observation trials and four positive imagery generation trials). The entire experiment consisted of two sessions. The order of the picture observation and positive imagery generation trials was randomised. The words and pictures used in the picture observation and positive imagery generation conditions were also randomised for each participant. The explanation of the experimental procedure was repeated until the participants thoroughly understood the task, and practice trials were conducted before the experimental trials.

### 2.5. fMRI Data Acquisition and Data Analysis

#### 2.5.1. fMRI Data Acquisition

A whole-body 1.5 T Signa Echo-Speed scanner (General Electric, Inc. Chicago, IL, USA) was used to acquire high-resolution spin echo T1-weighted anatomical images (20 axial slices, matrix size = 256 × 256, TR = 500 ms, FOV = 240 × 240, voxel size = 0.93 mm isotropic) and gradient echo echo-planar T2*-weighted images with a BOLD contrast (20 axial slices, matrix size = 64 × 64, flip angle = 90°, TR = 3000 ms, TE = 40 ms, FOV = 240 × 240 mm, voxel size = 3.75 mm isotopic, slice thickness = 4 mm, and slice gap = 0.8 mm). A total of 314 scans were acquired per participant (157 volumes × 2 sessions). Brain activity was measured throughout the session.

#### 2.5.2. fMRI Data Analysis

**Figure 2:**
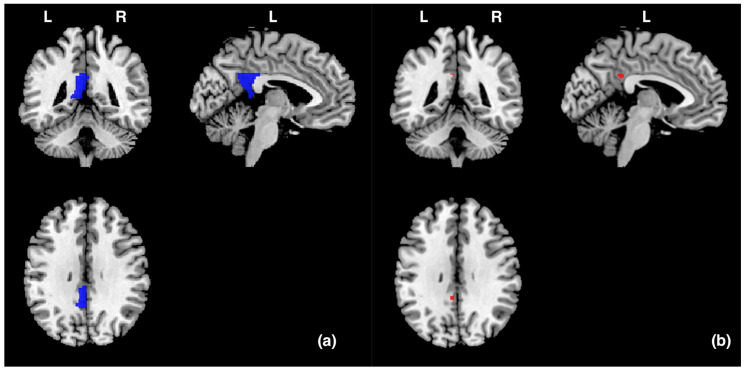
(**a**) The blue areas represent the ROI (the left PCgG) in this study, and the size was 3704 mm^3^ (463 voxels). (**b**) The red areas represent the areas (−4, −44, 32) in the left posterior cingulate gyrus that were significantly activated during picture observation compared to during positive imagery generation. L and R indicate left and right hemispheres, respectively.

Data were analysed via statistical parametric mapping (SPM8, www.fil.ion.ucl.ac.uk/spm/software/spm8, accessed on 8 January 2024, MATLAB version R2011b) using a general linear model. All functional volumes were realigned to each participant’s first volume to correct for head motion, which was spatially normalised to the Montreal Neurological Institute (MNI) brain template. Each voxel was resampled to 2 × 2 × 2 mm and smoothed with an 8 mm full-width-at-half-maximum Gaussian kernel. Functional data were analysed using a block design. For a group analysis, a random effects analysis was performed based on the general linear model. Each block was defined as a regressor of interest: picture observation, positive imagery generation, relax. A high-pass filter with a cut-off period of 128 s was used to remove low-frequency noise. We predefined the left PCgG as a region of interest (ROI) and prepared templates using the WFU PickAtlas tool 3.0.5 [33,34] according to an Automatic Anatomical Labeling (AAL) atlas previously reported in [35] (Figure 2). And we performed small volume correction (SVC) analyses on the ROI. We investigated whether stronger activity occurred in the left PCgG during picture observing (20 s) in the picture observation condition compared to imagery generation while seeing a picture (20 s) in the positive imagery generation condition. Brain activity during picture observation and imagery generation was averaged for each condition and participant. Brain activity during picture observation in the picture observation condition (or during positive imagery generation in the positive imagery generation condition) was the average activity of each participant over 8 trials (2 trials for each Gaussian filter of 0, 4, 8, and 12). As described above, although the main aim of this experiment was to compare brain activity during picture observation − during positive imagery generation, we also investigated whether stronger activities occurred in the left PCgG during positive imagery generation compared to during relaxation (20 s) in the baseline condition and during picture observation compared to during relaxation (20 s) in the baseline condition. In the present study, it was assumed that the left PCgG would be more active in a condition in which the suppression of imagery generation works more strongly (i.e., the picture observation condition) than in a condition in which it does not work (or only weakly works) (i.e., the positive imagery generation condition). However, it is also possible that the brain activity that occurs in such conditions reflects cognitive activities other than the suppression of imagery generation. For example, it may reflect visual perceptual and emotional processing, which may occur in the picture observation and positive imagery generation conditions. If this is the case, then stronger left PCgG activity should also occur in the positive imagery generation and picture observation conditions than in the baseline condition, in which positive emotion and visual perceptual processing would not occur. Therefore, we decided to compare brain activity during positive imagery generation − during relaxation in the baseline condition and during picture observation − during relaxation in the baseline condition. The baseline condition that was compared with the activation conditions (picture observation and positive imagery generation) was during relaxation in the baseline condition immediately before each activation condition. Statistical significance was set at *p* < 0.05, and the family-wise error (FWE) was corrected at the voxel level within small volume correction. In addition, the time course of the MRI signal was calculated using Marsbar [36].

## 3. Results

### 3.1. Brain Activity in Left Posterior Cingulate Gyrus

In order to identify the specific brain area involved in the suppression of imagery generation, we measured the brain activity in the left PCgG, whose activity was expected to be significantly greater during picture observation in the picture observation condition than during imagery generation in the positive imagery generation condition. As shown in Table 1 and Figure 2, significant activity (−4, −44, 32) was found (*p* (FWE) = 0.016).

In addition, brain activity was measured in the left PCgG during positive imagery generation − during relaxation in the baseline condition, and during picture observation − during relaxation in the baseline condition. No significant activity was found in either comparison.

### 3.2. Relationship between MRI Signal Changes in the Activated Area (−4, −44, 32) and the Participants’ Subjective Richness Ratings of the Pictures

If the activated brain area in the left PCgG plays a role in the perception-driven suppression of imagery generation, then there would be a correlation between the richness ratings, which are subjective assessments of the clarity of the presented pictures, and the activation levels in the activated brain area, such that increased activity in the activated brain area would be associated with a higher level of richness. Therefore, the correlation between the activity in the activated brain area and the richness ratings was examined. Each participant completed eight trials in the picture observation condition, and eight richness ratings were obtained for each participant, from which the mean richness rating for each participant was calculated. We calculated the correlation coefficient between the mean richness ratings and brain activity during picture observation for each of the 16 participants.

**Figure 3:**
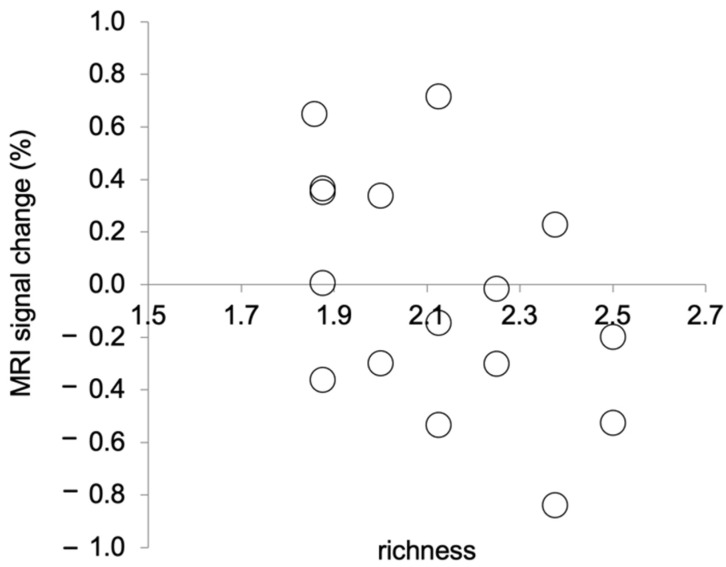
Correlation between MRI signal changes at (−4, −44, 32) during picture observation and richness ratings. A lower richness rating means that the presented picture was perceived as clearer.

We calculated the mean changes in the MRI signal as follows: First, we calculated the MRI signals in the brain area examined in this study using Marsbar. Then, we calculated the changes in the MRI signal during picture observation relative to those during relaxation. Relaxation in the baseline condition lasted for 20 s before the picture observation, which also lasted for 20 s. We calculated changes in the MRI signal during picture observation in the picture observation condition relative to those during relaxation in the most recent baseline condition. In this experiment, the TR duration was 3 s. Thus, the MRI signal data were recorded 6–7 times each during relaxation and during picture observation. The data from only six scans from the beginning to the end of an activity were analysed. These six scans were averaged for each activity. The MRI signal change was then calculated for the averaged MRI signal during picture observation relative to that during relaxation. The mean percentage change in the MRI signal and the mean richness score for the picture observation condition were calculated for each participant. That is, the following means were calculated for each participant across the eight picture observation trials: percentage change in the MRI signal and richness score. Shapiro–Wilk tests were performed on the mean percentage change in the MRI signal and the mean richness scores to test for normality. As a result, Spearman’s rank correlation was calculated, as the mean richness scores were not normally distributed (*p* = 0.06). There was a significant negative correlation between the mean percentage change in the MRI signal and the mean richness score (*r_s_* (14) = −0.50, *p* = 0.047, two tails, Figure 3). This result indicates that the stronger the activation of the brain area in the left PCgG, the clearer the recognition of the presented picture.

### 3.3. Relationship between MRI Signal Changes in the Activated Area (−4, −44, 32) and the Vividness of Imagery

Although a previous study suggests that there is no such correlation, if the activated brain area in the left PCgG were to play a role in suppressing the generation of any type of mental imagery, then there would be a correlation between the vividness of the imagery and the activity in the brain area, such that increased activity in the brain area would be associated with a reduced vividness of the positive imagery generated by the participants in this experiment. Therefore, the correlation coefficient between the MRI signal changes in the cluster containing (−4, −44, 32) and the subjective vividness of the imagery was calculated. The mean change in the MRI signal was calculated during positive imagery generation relative to that during relaxation in the most recent baseline condition. The method used to calculate the mean percentage signal change was identical to that described in Section 3.2. The mean subjective rating of the imagery vividness was calculated for each participant as follows: Each participant took part in eight positive imagery generation trials, and eight imagery vividness ratings were obtained for each participant. The mean of the imagery vividness ratings from these eight trials was taken as the mean imagery vividness rating for each participant. As there were 16 participants in this study, the mean imagery vividness ratings and brain activity during positive imagery generation for each participant resulted in 16 data points. The correlation coefficient was calculated between the two variables. The mean percent signal change and mean imagery vividness ratings were calculated for each participant, and a correlation analysis was performed on the two variables. A Pearson’s correlation coefficient was calculated because the Shapiro–Wilk tests conducted on the mean percentage change in the MRI signal and the mean imagery vividness ratings did not reject the null hypothesis, indicating that both variables were normally distributed. The results indicate that there is no significant correlation between the variables (*r* (14) = −0.07, *p* > 0.10).

## 4. Discussion

This study attempted to identify the brain regions involved in the perception-driven suppression of imagery generation. A previous study suggested that the left PCgG is involved in the suppression of negative imagery generation. If the left PCgG were involved in the suppression of imagery in general, it would be activated not only during the formation of negative imagery but also during other activities that require the suppression of the formation of mental imagery. It has been shown that the processes of visual perception and visual imagery formation are in a competitive relationship. That is, it is expected that at least some regions of the left PCgG would be activated during the observation of visual perceptual stimuli because the formation of mental imagery would be suppressed while visual perceptual images are actively observed.

In this study, part of the left PCgG was more active during the picture observation condition, during which the suppression of perception-driven imagery generation was considered to be stronger than during the positive imagery generation condition. Furthermore, there was a significant negative correlation between the activity in the left PCgG and the perceptual richness ratings. More specifically, the higher the activity in the brain area, the lower the richness ratings (i.e., the clearer the picture). Additionally, the lower the activity in the brain area, the higher the richness ratings. If we assume that the left PCgG plays a role in suppressing the generation of mental imagery, then we can offer a consistent explanation for these results. That is, when the brain area concerned was more strongly activated and the suppression of imagery generation function was working more strongly, visual perceptual processing was more activated because competitive mental imagery generation did not occur. Therefore, a visual object was perceived as clearer when the relevant brain area was more strongly activated. These results suggest that the left PCgG plays a role in the suppression of imagery generation.

Motoyama and Hishitani (2016) previously demonstrated the imagery suppression mechanism in a research context slightly different to that used in the present study [24]. They showed significantly increased activation in a part of the left PCgG during negative imagery generation compared to during positive imagery generation. Their finding indicates that the activity in the left PCgG increased during negative imagery generation when the emotion-driven suppression of imagery generation was stronger. From a perspective different to that of Motoyama and Hishitani (2016) [24], the result of the current study shows that the left PCgG plays a role in the suppression of imagery generation. However, the activated brain coordinates found in this study do not perfectly match the area involved in the suppression of mental imagery generation shown in [24]. The peak Talairach coordinates of the activated brain area in [24] were (−18, −53, 30), and the difference in the peak coordinates between this study and those in [24] is about 18.5 mm, suggesting that the perception-driven and emotion-driven suppression of imagery generation may be caused by different mechanisms. Since this possibility is only based on the results of [24] and the present study, it needs to be examined in further experiments.

The present study found that the left PCgG was more strongly activated in the picture observation condition than in the positive imagery condition. This finding provides evidence for the notion that the left PCgG plays a role in the perception-driven suppression of imagery generation. However, if an increase in visual perceptual processing could lead to an increase in PCgG activity, then the result could reflect the visual perceptual information processing activity that occurs in the PCgG. This interpretation can be ruled out for two reasons. First, if the activity observed in the left PCgG in this experiment reflected visual perceptual processing, then the activity in this brain area would also have been produced in the picture observation condition—in the baseline condition. However, no such brain activity was found. Second, a previous study suggested the possibility that the PCgG does not play a role in the processing of visual perceptual information, such as in the observation of a picture in this experiment. It has been postulated that PCgG activity is reduced when paying attention to the outside world [37,38,39], suggesting that PCgG activity is reduced when actively looking at pictures, as in the task used in this experiment. In addition, it has been suggested that similar levels of activity in the PCgG occur during eye closure and visual processing [38], and no significant change in blood flow to the PCgG has been shown between passive fixation and eye closure [39]. Therefore, it is very likely that the visual perceptual processing required in this experiment and the activity in this brain region are independent. Furthermore, the results of a study conducted by Uddin, Clare Kelly, Biswal, Castellanos, and Milham (2009), which showed a negative correlation between PCgG activity and the extrastriate visual areas involved in visual perceptual processing [40], suggest that it is very unlikely that the PCgG is involved in goal-directed visual perceptual information processing. Therefore, the result obtained in this study, i.e., the higher activation of the left PCgG in the picture observation condition than in the positive imagery generation condition, most likely reflects the perception-driven suppression of mental imagery generation rather than visual perceptual information processing.

If the left PCgG plays a role in suppressing the generation of any kind of mental imagery, then this implies that greater activity of the imagery suppression function, reflected in increased activity in the left PCgG, would lead to a reduction in the vividness of imagery. However, no such relationship was found in this experiment. This suggests that the perception- and emotion-driven suppression of imagery generation did not occur during the generation of positive imagery. Motoyama and Hishitani (2016) previously confirmed that the vividness of negative imagery varies with the activity of this brain area [24], and, consequently, the above fact suggests that the vividness of the positive imagery was influenced by other factors. It has been suggested that the vividness of imagery is not determined by a single function [1]. Therefore, future studies are needed to investigate how the vividness of positive imagery is determined, in addition to the imagery suppression function demonstrated in this study.

The left PCgG activity observed in this experiment may reflect other types of processing rather than the suppression of imagery generation. For example, it is likely that positive emotions were evoked in both the positive picture observation and positive imagery generation conditions. Although the participants’ task in the positive picture observation condition was to observe positive pictures without interference, in the positive imagery generation condition, the participants simultaneously saw neutral pictures unrelated to the imagery object during positive imagery generation. That is, the evocation of positive emotions might be attenuated in the positive imagery generation condition compared to the case wherein positive imagery is simply generated without seeing a picture. A previous study also suggested that the PCgG is involved in the processing of positive emotions [41]. In other words, it could be considered that the activity in the left PCgG was stronger in the positive picture observation condition than in the positive imagery generation condition because the positive emotions evoked in the positive picture observation condition were greater than those evoked in the positive imagery generation condition despite the suppression of imagery generation. However, it is unlikely that the activity detected in the left PCgG in this experiment reflected positive emotion processing. It is likely that positive emotion processing occurred more in the picture observation and positive imagery generation conditions than in the baseline condition, which did not evoke emotions. That is, if PCgG activity reflected positive emotion processing, then this activity would also have been observed in the picture observation condition − in the baseline condition and in the positive imagery generation condition − in the baseline condition. In this experiment, no significant activity was found in either condition. These results suggest that the PCgG activity observed in this experiment does not reflect positive emotional processing.

## 5. Limitations and Implications of this Study

As the number of participants in this study may not be large enough, it would be desirable to conduct a similar experiment with a larger number of participants to see whether the results of this experiment can be replicated.

Furthermore, we believe that this study can be further improved by repeating similar experiments, not only with more participants but also with more accurate MRI equipment, such as 3T. The brain region responsible for perception-driven imagery suppression shown in the present study and the brain region responsible for emotion-driven imagery suppression shown in previous studies are both located in the left PCgG, but they do not completely overlap. In the future, it would be desirable to clarify whether the two functions are the same or different imagery suppression functions. By increasing the number of participants and using more accurate MRI machines to measure more precisely whether the brain regions activated during the two functions are different, it may be possible to investigate whether the perception-driven and emotion-driven functions are the same imagery suppression mechanism.

## 6. Conclusions

The novel finding of this study is the demonstration of the existence of a neural basis for the suppression of imagery generation, achieved using an approach that is different from that used in a previous study. In the previous study, significant activity in the left PCgG was found during negative imagery generation, where imagery generation was suppressed, compared to during positive imagery generation, where imagery generation was not suppressed. This finding suggests that the left PCgG is involved in the suppression of imagery generation. In this study, we investigated whether significant activity in the left PCgG also occurs during visual perceptual processing in which imagery generation is suppressed, which differed from the previous study. As a result, it was found that significant activity in the left PCgG occurred during visual perceptual processing compared to during positive imagery generation; thus, using a different approach to the previous study, this experiment provides further evidence that the left PCgG is involved in the suppression of imagery generation.

## Figures and Tables

**Table 1:** Areas of significant activation observed during picture observation compared to during positive imagery generation.

Brain Area Brodman Area (BA)	Number of Voxels in Cluster	Voxel−Level *p* Value FWE−Corrected	Voxel−Level *p* Value Uncorrected	T Value at Local Maximum	MNI Coordinates		
					x	y	z
Posterior Cingulate Gyrus (BA31)	4	0.016	0	4.71	−4	−44	32

## Data Availability

The data used in this current study is available from the corresponding author upon reasonable request.

## Notes

1 Previous research has not only shown that negative imageries are less vivid than positive ones, but also that negative autobiographical memories are less likely to be recalled. For example, involuntary autobiographical memories in everyday life have been shown to be more positive and neutral than those with negative emotions [42], and voluntary autobiographical memories are also more positive than negative, despite being weaker than involuntary autobiographical memories [43]. When participants in their 20s to 90s were asked about their involuntary autobiographical memories, it was found that they had more happy and mixed emotional memories than sad ones [44].

2 Intrusive imagery, a type of negative imagery that is a clinical symptom of PTSD flashbacks, depression, and anxiety, has been shown to be extremely vivid [45]. Motoyama and Hishitani (2016) described negative imagery as being more vague than positive imagery [24]. However, the negative imagery that they focused on was not intrusive imagery but rather imagery that was intentionally generated by healthy individuals. Therefore, the vague negative imagery described by Motoyama and Hishitani (2016) does not include intrusive imagery.

3 In this experiment, we used the term “vividness” when participants rated the vividness of the generated imageries in the positive imagery condition, and we used the term “richness” when participants rated the clarity of the presented picture in the picture observation condition.

## Appendix A

The pictures presented in the picture observation condition and the imagery object in the positive imagery generation condition. Half of the pictures were presented in the picture observation condition for one participant, and the words corresponding to these pictures were used as imagery objects in the positive imagery generation condition for the other participant. The eight pictures presented in the picture observation condition consisted of two 0 mm, two 4 mm, two 8 mm, two 12 mm of Gaussian filter effects.

## Appendix B

The pictures presented in the positive imagery generation condition. Half of the pictures were presented in one participant’s positive imagery generation condition and the other half in the other participant’s positive imagery generation condition. The eight pictures presented to each participant consisted of two 0 mm, two 4 mm, two 8 mm, two 12 mm of Gaussian filter effects.

## References

[1] Hishitani S., Miyazaki T., Motoyama H. (2011). Some mechanisms responsible for the vividness of mental imagery: Suppressor, closer, and other functions. J. Ment. Imag..

[2] Kosslyn S.M. (1980). Image and Mind.

[3] Kosslyn S.M., Thompson W.L., Ganis G. (2006). The Case for Mental Imagery.

[4] Pearson D.G., Andrade J. (2001). Imagery and the visuo-spatial sketchpad. Working Memory in Perspective.

[5] Pearson D.G., Logie R.H., Gilhooly K.J. (1999). Verbal representation and spatial manipulation during mental synthesis. Eur. J. Cogn. Psychol..

[6] Pearson D.G., Logie R.H., Green C. (1996). Mental manipulation, visual working memory, and executive processes. Psychol. Beitr..

[7] Kosslyn S.M. (1994). Image and Brain: The Resolution of the Imagery Debate.

[8] Brooks L.R. (1967). The suppression of visualization by reading. Q. J. Exp. Psychol..

[9] Chang S., Lewis D.E., Pearson J. (2013). The functional effects of color perception and color imagery. J. Vis..

[10] Craver-Lemley C., Arterberry M., Reeves A. (1997). Effects of imagery on vernier acuity under conditions of induced depth. J. Exp. Psychol. Hum. Percept. Perform..

[11] Craver-Lemley C., Arterberry M., Reeves A. (1999). “Illusory” illusory conjunctions: The conjoining of features of visual and imagined stimuli. J. Exp. Psychol. Hum. Percept. Perform..

[12] Craver-Lemley C., Reeves A. (1987). Visual imagery selectively reduces vernier acuity. Perception.

[13] Craver-Lemley C., Reeves A. (1992). How visual imagery interferes with vision. Psychol. Rev..

[14] Ishai A., Sagi D. (1997). Visual imagery: Effects of short- and long-term memory. J. Cogn. Neurosci..

[15] Keogh R., Pearson J. (2011). Mental imagery and visual working memory. PLoS ONE.

[16] Keogh R., Pearson J. (2014). The sensory strength of voluntary visual imagery predicts visual working memory capacity. J. Vis..

[17] Keogh R., Pearson J. (2017). The perceptual and phenomenal capacity of mental imagery. Cognition.

[18] Kwok E.L., Leys G., Koenig-Robert R., Pearson J. (2019). Measuring Thought-Control Failure: Sensory Mechanisms and Individual Differences. Psychol. Sci..

[19] Reeves A. (1981). Visual imagery lowers sensitivity to hue-varying, but not to luminance-varying, visual stimuli. Percept. Psychophys..

[20] Reeves A., Craver-Lemley C. (2012). Unmasking the Perky effect: Spatial extent of image interference on visual acuity. Front. Psychol..

[21] Segal S.J., Fusella V. (1970). Influence of imaged pictures and sounds on detection of visual and auditory signals. J. Exp. Psychol..

[22] Sherwood R., Pearson J. (2010). Closing the mind’s eye: Incoming luminance signals disrupt visual imagery. PLoS ONE.

[23] Kosslyn S.M. (1987). Seeing and imaging in the cerebral hemispheres: A computational approach. Psychol. Rev..

[24] Motoyama H., Hishitani S. (2016). The brain mechanism that reduces the vividness of negative imagery. Conscious. Cogn..

[25] Bywaters M., Andrade J., Turpin G. (2004). Intrusive and non-intrusive memories in a non-clinical sample: The effects of mood and affect on imagery vividness. Memory.

[26] Hertel P.T., Parks C. (2002). Emotional episodes facilitate word recall. Cogn. Emot..

[27] Hishitani S. (1995). Toward a deeper understanding of vividness: Some points inspired from McKelvie’s Article. J. Ment. Imag..

[28] Segal S.J., Fusella V. (1971). Effect of images in six sense modalities on detection of visual signal from noise. Psychon. Sci..

[29] Oldfield R.C. (1971). The assessment and analysis of handedness: The Edinburgh inventory. Neuropsychologia.

[30] Miyazaki T., Motoyama H., Hishitani S. (2003). Emotional values for nouns and epithets: Standardization on the positive-negative dimension. Jpn. J. Ment. Imag..

[31] Nova Development (2003). Art Explosion 750,000 for Macintosh.

[32] Hishitani S., Motoyama H., Hishitani S. (2009). The trial manufacture of switch box for fMRI experiment. Hardware and Software for Psychology Laboratories.

[33] Maldjian J.A., Laurienti P.J., Burdette J.H. (2004). Precentral gyrus discrepancy in electronic versions of the Talairach atlas. NeuroImage.

[34] Maldjian J.A., Laurienti P.J., Kraft R.A., Burdette J.B. (2003). An automated method for neuroanatomic and cytoarchitectonic atlas-based interrogation of fMRI data sets. NeuroImage.

[35] Tzourio-Mazoyer N., Landeau B., Papathanassiou D., Crivello F., Etard O., Delcroix N., Mazoyer B., Joliot M. (2002). Automated anatomical labeling of activations in SPM using a macroscopic anatomical parcellation of the MNI MRI single-subject brain. Neuroimage.

[36] Brett M., Anton J.L., Valabregue R., Poline J.B. Region of interest analysis using an SPM toolbox. Proceedings of the 8th International Conference on Functional Mapping of the Human Brain.

[37] Buckner R.L., Andrews-Hanna J.R., Schacter D.L. (2008). The brain’s default network: Anatomy, function, and relevance to disease. Ann. New York Acad. Sci..

[38] Greicius M.D., Krasnow B., Reiss A.L., Menon V. (2003). Functional connectivity in the resting brain: A network analysis of the default mode hypothesis. Proc. Natl. Acad. Sci. USA.

[39] Raichle M.E., MacLeod A.M., Snyder A.Z., Powers W.J., Gusnard D.A., Shulman G.L. (2001). A default mode of brain function. Proc. Natl. Acad. Sci. USA.

[40] Uddin L.Q., Clare Kelly A.M., Biswal B.B., Castellanos F.X., Milham M.P. (2009). Functional connectivity of default mode network components: Correlation, anticorrelation, and causality. Hum. Brain Mapp..

[41] Aldhafeeri F.M., Mackenzie I., Kay T., Alghamdi J., Sluming V. (2012). Regional brain responses to pleasant and unpleasant IAPS pictures: Different networks. Neurosci. Lett..

[42] Berntsen D. (1996). Involuntary autobiographical memories. Appl. Cogn. Psychol..

[43] Berntsen D. (1998). Voluntary and involuntary access to autobiographical memory. Memory.

[44] Berntsen D., Rubin D.C. (2002). Emotionally Charged Autobiographical Memories Across the Life Span: The Recall of Happy, Sad, Traumatic, and Involuntary Memories. Psychol. Aging.

[45] Çilli S., Stopa L. (2015). Intrusive mental imagery in psychological disorders: Is the self the key to understanding maintenance?. Front. Psychiatry.

